# Enhanced Microwave Commutation Quality Factor of Tunable Capacitors Based on SrTiO_3_ Thin Films

**DOI:** 10.3390/molecules30234593

**Published:** 2025-11-29

**Authors:** Andrei Tumarkin, Alexey Bogdan, Eugeny Sapego, Oleg Korepanov, Artem Karamov

**Affiliations:** Department of Physical Electronics and Technology, Saint Petersburg Electrotechnical University “LETI”, 197022 Saint Petersburg, Russia

**Keywords:** strontium titanate film, oriented growth, alumina substrate, microwave applications

## Abstract

Thin films of strontium titanate were grown on a polycrystalline aluminum oxide substrate using magnetron sputtering. These films exhibit high structural quality and nonlinear properties, which make them promising for microwave applications. Planar capacitors based on SrTiO_3_ films demonstrated a tunability of 1.65 with a microwave Q-factor of at least 110 in the entire range of control voltages without deterioration of losses, and a slow capacitance relaxation level no more than 4%, which is significantly better than currently published data for planar ferroelectric elements. This is the first successful attempt to create a planar SrTiO_3_ capacitor on an alumina, which demonstrates a commutation quality factor CQF of 3300 at microwaves.

## 1. Introduction

Ferroelectric (FE) materials are of interest for microwave electronics due to their anomalously high dielectric permittivity. The nonlinear dependence of the FE permittivity on an external electric field allows the implementation of microwave devices with electrically controlled characteristics, such as varactors, phase shifters, tunable filters, delay lines, phased array antennas, etc. [1,2,3]. For practical applications, three characteristics of microwave FE elements, determined by the properties of the material, play a primary role. The first characteristic is tunability, i.e., the scale of change in permittivity under the action of an external field. Today, studies have been published in which film FE capacitive elements based on solid solutions of barium and strontium titanates BaSrTiO_3_ (BST) demonstrate a change in permittivity ε by 13 [4] and even almost 100 times [5] in fields on the scale of 100 V/μm. The maximum values of tunability of FE film structures are achieved either in the metal–dielectric–metal (MDM) design, where a high control field strength is easily provided [6], or in strained films grown on substrates of dysprosium and samarium scandates [4,5,7]. The second important characteristic is the long-term stability of ferroelectric elements. The work of Astafiev et al. demonstrated that FE capacitors can withstand 10^7^ switching cycles without degradation [8]. This corresponds to 5–6 years of continuous operation of a phase shifter in a microwave phased array antenna. Other studies demonstrate that the FE capacitors reliably withstand 10^10^ switching cycles without degradation of their capacitance, tunability, and quality factor [9,10,11]. Based on these studies, ferroelectric capacitive structures have great potential for long-term applications. The third important characteristic is the microwave losses of the element, which include losses in the ferroelectric and in the electrodes and, depending on the frequency, determine the attenuation of the signal during the change in permittivity. The nature of ferroelectric materials is such that these characteristics are interrelated, and an increase in the tunability of the FE device, as a rule, leads to an increase in losses. A compromise can be found by using the commutation quality factor (CQF) introduced by Vendik et al. and taking into account these characteristics simultaneously [12]. Thus, at room temperature, the commutation quality factor of elements with a tunability of 13 is 2750 at a frequency of 2.4 GHz [4], and for elements with a tunability of 85 is 2800 at 100 kHz [5], due to high losses. Thus, high tunability does not determine the successful use of FE elements in microwaves. Moreover, for many applications, tunability of one and a half to two times is sufficient [3], while high losses can only be compensated for by repeated signal amplification, which is not always acceptable. There are microwave applications in which not only low but also non-degrading losses under the application of a control field are critical. One example is a radiating phased array antenna with ferroelectric phase shifters. These phase shifters are located directly in front of the antenna’s radiating elements, and there is no way to re-amplify the signal after losses in the phase shifter. In this case, the quality of the transmitted signal will directly depend on the losses in the phase shifter, and the deterioration of the FE Q-factor when applying a control voltage will lead to signal degradation. As an example, works [13,14] can be given where CQF of about 10^6^ at 1 MHz, and 7 × 10^3^ at 1 GHz are demonstrated, respectively, but the Q-factor deteriorates by an order of magnitude or more when a control voltage is applied. Thus, the search for nonlinear dielectrics that exhibit acceptable tunability with low and non-degrading microwave losses is a challenge today.

As an alternative to barium strontium titanate, the most studied ferroelectric for microwave applications, we consider pure strontium titanate SrTiO_3_ (STO). Potential advantages of STO over BST at microwaves are as follows: (a) a significantly higher Q-factor, determined by the absence of spontaneously polarized regions [15]; (b) a fast response of the material to external influences due to a minimal number of structural defects [16]; (c) temperature stability due to the paraelectric state of STO down to cryogenic temperatures. A challenge in attempts to use film strontium titanate for tunable applications is obtaining films with an acceptable level of change in permittivity under the influence of a control field at room temperature. For small-signal applications, this problem is solved by using a plane-parallel MDM design of the capacitive element by reducing the gap size of the capacitor and, consequently, increasing the value of the control field. The disadvantages of MDM capacitive elements include limitations in handling high-power signals, technological difficulties in implementing multilayer structures with a low-losses lower conducting electrode, and difficulties in implementing large nominal capacities. These disadvantages do not apply to planar structures on a dielectric substrate; they can be used to implement low-cost distributed structures for frequency ranges above 30 GHz [17] and capacitive elements for high-power applications [18]. In any case, the high nonlinear properties and quality factor of STO elements are directly related to the structural perfection of the films.

Today, oriented STO films have been obtained on rather expensive single-crystal substrates of magnesium oxide [19], sapphire [20], LaAlO_3_ [21], and SrTiO_3_ [22]. It has been shown that the main technological parameters influencing the structural properties of strontium titanate films are the substrate material and temperature [18,19], as well as the composition and pressure of the working gas [23,24]. Several characteristic features inherent in the studies of STO films should be noted. In most cases, the structure of strontium titanate is studied either on structurally matched single-crystal substrates [21,22] with a platinum or SrRuO_3_ sublayer. For example, the work of Sangle et al. [22] considers STO MDM capacitors on a strontium titanate substrate with SrRuO_3_ bottom electrodes and Pt top electrodes. The capacitors obtained in this work demonstrate a commutation quality factor of 3300 at a frequency of 1 MHz. Since both losses and CQF depend on frequency, it can be assumed that at microwaves, the losses of these capacitors will increase significantly, and the CQF will deteriorate. Furthermore, the electrodes will also significantly contribute to losses at microwaves due to their relatively low electrical conductivity. A second important aspect of the work is the use of a strontium titanate substrate with a dielectric permittivity of about 300. This will significantly complicate the matching of the capacitor with other elements of the microwave circuit.

From the microwave application standpoint, the growth of oriented STO films on polycrystalline Al_2_O_3_ (alumina) substrates is of significantly greater interest due to its mechanical and microwave properties comparable to monocrystalline sapphire, but with a significantly lower substrate cost. Therefore, the aim of this study is to investigate the growth processes of STO films depending on process conditions to obtain high-quality layers on an alumina dielectric substrate, as well as to investigate the structural properties of the films and the electrical characteristics of planar capacitive elements based on them for their subsequent use in tunable microwave devices.

## 2. Results and Discussion

### 2.1. Structural Characterization

The composition and pressure of the working gas, in which the target is sputtered and the sputtered atoms are transported to the substrate, affect the thermalization length of the sputtered particles and the flux density of atoms arriving at the substrate, which, along with the deposition temperature, determines the mechanisms of film nucleation and growth. Studies of the properties of strontium titanate films depending on the deposition temperature [18] show that predominantly oriented STO films are formed on an alumina at growth temperatures above 800 °C. At high substrate temperatures, the nucleation of oriented islands becomes energetically favorable, which determines the subsequent formation of an (h00) oriented layer with a minimum content of secondary phases. Thus, we expect that the deposition of STO films at high substrate temperatures and in a gas environment optimal in composition and pressure potentially allows for the production of defect-free, highly oriented layers.

**Figure 1:**
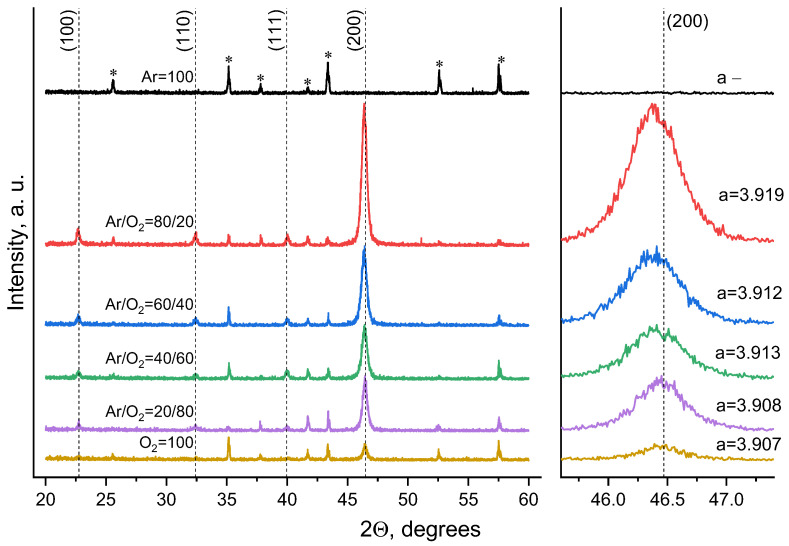
Diffraction patterns of STO films deposited on an alumina at different oxygen contents in the Ar:O_2_ gas mixture, *—the substrate reflections.

Figure 1 shows the diffraction patterns of STO films deposited on an alumina substrate at different oxygen contents in the Ar:O_2_ gas mixture at a total system pressure of 3 Pa. The asterisks indicate the substrate reflections, and the vertical dashed lines indicate the positions of the STO reflections according to PDF 35-734. The XRD data allow us to conclude that the angular positions of the diffraction peaks correspond to the structure of strontium titanate, and the interplanar distances in the direction normal to the STO film surface depend on the oxygen content in the working gas. Films deposited at high oxygen concentrations exhibit a polycrystalline unstrained structure with the main crystalline phase (h00). As the oxygen content decreases, the X-ray reflections shift toward smaller angles, which can be compared with an increase in oxygen deficiency in the films [21]. An analysis of the unit cell dimensions (see inset in Figure 1) suggests that films deposited in a gas mixture with a reduced oxygen content exhibit tensile stresses that increase the unit cell size (3.919 Å for the film deposited in an Ar:O_2_ 80/20 mixture compared to 3.905 Å for the single crystal). The most pronounced (h00) reflections are exhibited by films deposited in a gas mixture with an oxygen content of 20%.

**Figure 2:**
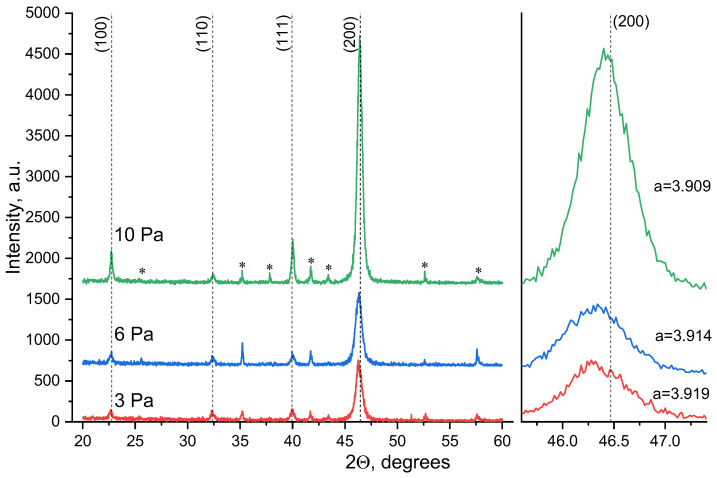
Diffraction patterns of STO films obtained at different gas pressures in an Ar:O_2_ 80/20 mixture, *—the substrate reflections.

In the search for process conditions that would allow the formation of STO films with minimal oxygen deficiency and unit cell distortions, in the next series of experiments, samples were deposited at different working gas pressures. Figure 2 shows the diffraction patterns of STO films deposited in an Ar:O_2_—80/20 gas mixture at working gas pressures of 3, 6, and 10 Pa. The graph shows that the film grown at a pressure of 10 Pa has a preferred orientation (h00) with minimal unit cell distortion compared to the other studied samples and to the single crystal. Increasing the working gas pressure during film deposition allows for several effects to be achieved. Firstly, it leads to the thermalization of all sputtered atoms of various masses near the target, after which they move in the diffusion mode and reach the substrate, being in thermodynamic equilibrium with the gas environment [25]. Secondly, high working gas pressure increases the number of oxygen atoms participating in film formation. Thirdly, increasing the pressure of the gas mixture reduces the flux density of sputtered atoms arriving at the substrate per unit time, which increases the adatom lifetime on the surface and creates conditions for oriented film growth [26] (the film growth rate under our conditions is 30 Å/min at a pressure of 3 Pa and 15 Å/min at 10 Pa). As a result, the growth of STO films at a working gas pressure of 10 Pa allows the formation of a predominantly oriented strontium titanate film on a structurally non-matched alumina with minimal oxygen defects compared to films deposited at lower pressures.

Summing up analyzing strontium titanate films using XRD, three factors can be identified. (A) In a film obtained in pure oxygen, the unit cell parameter *a* practically corresponds to the parameter of the bulk crystal. (B) With a decrease in the oxygen content in the gas mixture, a significant shift in the angular positions of X-ray reflections is observed toward smaller theta angles (Figure 1). (C) With increasing pressure of the Ar/O_2_ 80:20 gas mixture, the opposite situation is observed: the peak position shifts toward larger angles (Figure 2).

For multicomponent films, there are several reasons for the shift in the angular position of the X-ray reflection. First, this shift can be caused by mechanical stresses within the film. It should be noted that the shift in the reflection is affected only by stresses existing throughout the film volume, i.e., stresses of the first kind, characteristic of single-crystal films [27]. In a polycrystal, stresses within the crystallite, so-called stresses of the second kind, may be present. These stresses relax at grain boundaries, so they affect the shape of the X-ray peak but not its position. Furthermore, the influence of mechanical stresses along one axis can result in a tetragonal distortion of the cubic lattice, such as the appearance of a (200) and (002) doublet instead of a single peak. In our case, based on the XRD data, we can conclude that the studied films are not single-crystal and, furthermore, they lack tetragonal distortions of the unit cell. Secondly, the shift in the angular position of the reflection can be caused by changes in the component composition of the solid solution. Typical examples include barium–strontium titanate films with varying Ba/Sr ratios depending on the deposition conditions [28]. A third cause of the peak position shift is the distortion of the unit cell due to the formation of oxygen vacancies (V_O2_). This situation is typical for oxide films, and the number of vacancies depends on the gas environment and temperature at which the film is deposited. It is known that the presence of oxygen vacancies in the film leads to an increase in the unit cell size [29,30,31].

Thus, the assumption of the presence of oxygen vacancies in the films under study and the dependence of their number on film growth conditions is confirmed by X-ray diffraction data on the unit cell sizes. Films deposited in pure oxygen do not exhibit oxygen deficiency. As the O_2_ content in the gas mixture decreases, the film becomes depleted of oxygen and the unit cell parameter increases. Increasing the Ar/O_2_ mixture pressure leads to an increase in the oxygen partial pressure, which reduces the number of anion vacancies in the deposited layer. In this case, it can be expected that a predominantly oriented film with minimal oxygen deficiency will exhibit the best dielectric properties.

**Figure 3:**
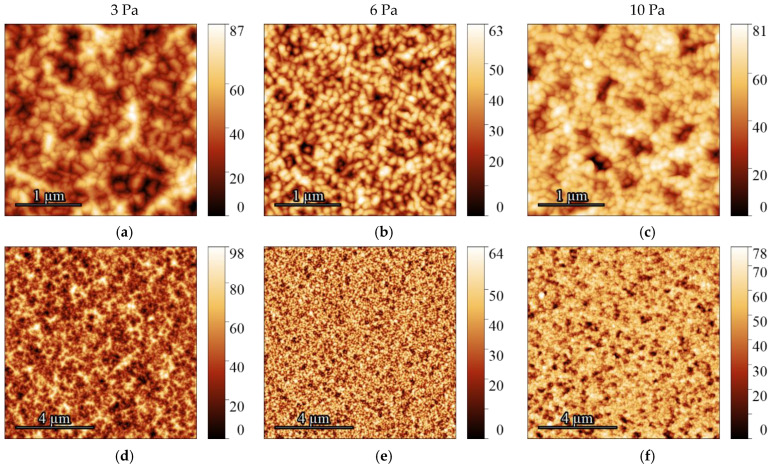
AFM images of STO films deposited on sapphire at oxygen pressures of 3, 6, and 10 Pa. Images are arranged left to right in order of increasing pressure. The top row (**a**–**c**) displays 3 × 3 μm scan areas; the bottom row (**d**–**f**) shows 10 × 10 μm scan areas. The vertical scale is represented in nm.

Figure 3 presents AFM images of the STO film surfaces deposited in an Ar:O_2_ (80/20) gas mixture at pressures of 3, 6, and 10 Pa. The film deposited at 3 Pa consists of misoriented crystallites with a size of 106 ± 63 nm and a root mean square (Sq) roughness of approximately 15 ± 7 nm. When the pressure is increased to 6 Pa, the resulting film exhibits a similar grain size of 108 ± 66 nm. While the misorientation of crystallites persists, the roughness increases to about 20 ± 14 nm. In contrast, the films deposited at the highest pressure of 10 Pa are characterized by a dense packing of oriented grains, measuring 113 ± 138 nm, with a reduced roughness of about 17 ± 7 nm. Based on this data, the pressure exerts a complex, non-monotonic influence on the film structure, with no clear linear correlation observed between pressure and parameters such as grain size or roughness. The grain size remains statistically unchanged, whereas the roughness reaches a maximum at the intermediate pressure of 6 Pa.

**Figure 4:**
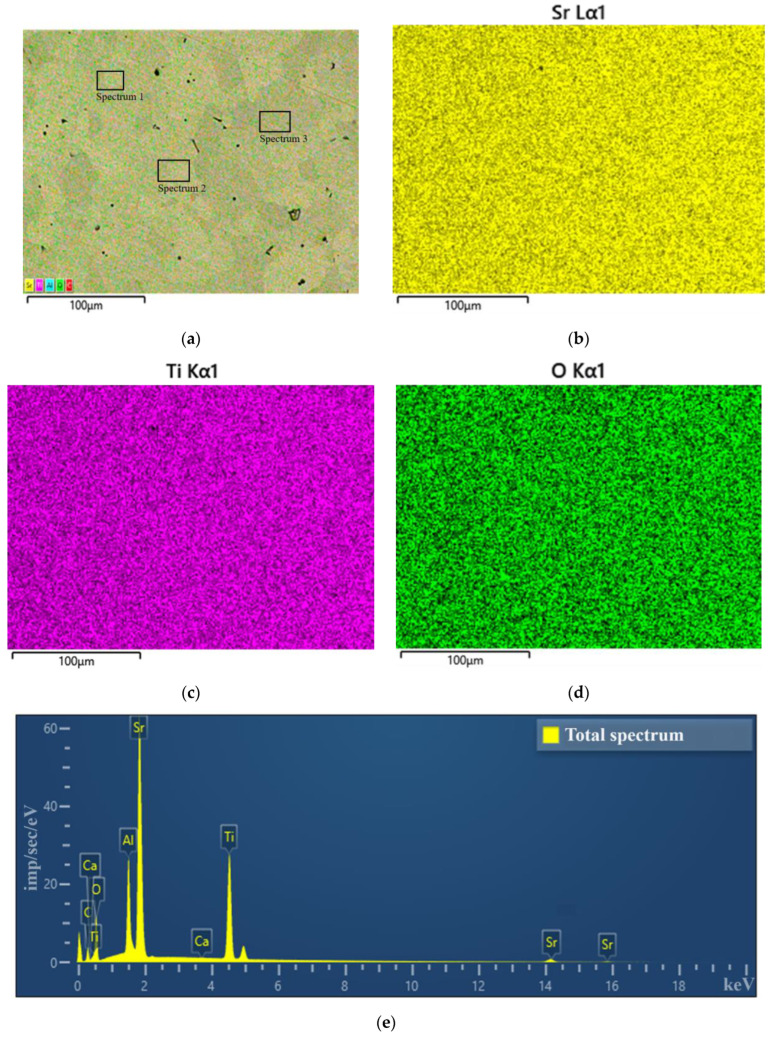
SEM images of the SrTiO_3_ film: (**a**)—fragment of the sample surface with indication of the areas where energy dispersive analysis was carried out; (**b**–**d**)—distribution of elements over the surface; (**e**)—EDS spectrum from SrTiO_3_ film.

Figure 4a shows a typical SEM image of the surface of a strontium titanate film on an alumina, indicating the areas where elemental analysis was performed. The film has a block structure characteristic of multicomponent layers deposited on a structurally inconsistent polycrystalline substrate. Figure 4b,d present data on the spatial distribution of the main chemical elements in the studied material. The results of energy-dispersive analysis of the films are presented in Figure 4d, and the average quantitative data of the elemental analysis are presented in Table 1. The samples exhibit a uniform distribution of elements over the surface area and the absence of significant amounts of foreign impurities. From the elemental analysis data, it can be concluded that the component composition of the sputtered target is stoichiometrically transferred to the film within the error limits of the method. The presence of a signal from aluminum from the substrate in the spectrum indicates the small thickness of the studied films.

Thus, the high deposition temperature and working gas composition of Ar:O_2_ 80/20 provide conditions for the oriented growth of defect-free strontium titanate films, which we expect to exhibit excellent electrical properties. To confirm this, planar capacitors were formed using films deposited in an Ar:O_2_ 80/20 gas mixture, and their electrical properties were studied.

### 2.2. Electrical Characterization

**Figure 5:**
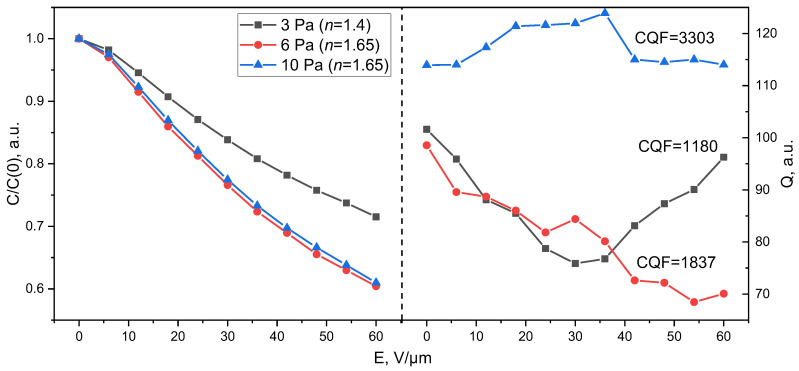
Normalized capacitance and quality factor of capacitors obtained at different working gas pressures.

Figure 5 shows the dependences of the capacitance normalized to the maximum value C(0 V) and the quality factor of planar STO capacitors on the control field strength. It is evident from the figure that the capacitors based on films deposited at a working gas pressure of 3 Pa demonstrate a tunability of 1.4 times (30%). Films deposited at high working gas pressures exhibit a less defective and less stressed structure, which correlates with an increase in tunability to *n* = 1.65 (40%). It should be noted that the thickness of the ferroelectric layer in a planar capacitor determines both tunability and losses. If the layer is thin, the substrate capacitance affects the parameters of the planar capacitor, reducing its tunability [32]. According to our preliminary data, the ferroelectric layer thickness that ensures maximum tunability of a planar capacitor is approximately 800 nm [33]. Therefore, we expect that increasing the film thickness in a planar capacitive structure will result in increased tunability due to the elimination of the substrate from the equivalent circuit of the planar capacitor. Figure 5 demonstrates a significant difference in the quality factor of capacitors based on films obtained at different gas mixture pressures, which is consistent with the differences in their structure. Comparison of the characteristics of the obtained structures with the currently published data on capacitors based on STO (best CQF data) and BST (best tunability data) films is presented in Table 2. It follows from the data in Figure 5 and Table 2 that the capacitive structures on an alumina obtained in this work demonstrate a promising quality factor in the microwave range [3]. Taking into account the relationship between the electrophysical parameters (tunability and dielectric losses) and the cost of manufacturing a planar capacitor compared to an MDM one, this result looks promising for microwave applications.

**Figure 6:**
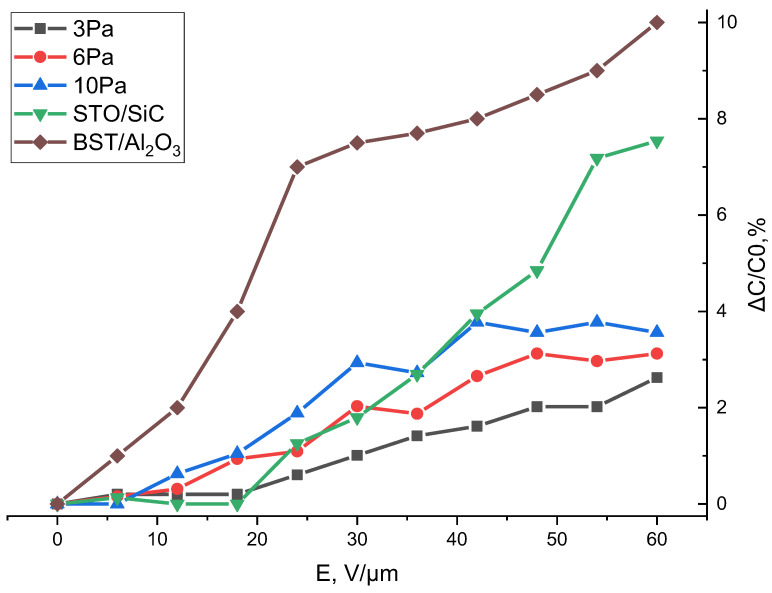
Response time parameter of capacitors obtained at different working gas pressures (STO/SiC data—[33], BST/Al_2_O_3_ data—[38]).

An important characteristic of microwave FE devices is the response speed of the permittivity to the application and, more importantly, removal of the control field. While the decrease in ε under the influence of the field typically occurs over times of the order of 10^−9^ s, the return of the permittivity to its initial value after the removal of the control action can occur significantly more slowly. This phenomenon, known as “slow relaxation”, is determined by charge injection from the electrodes, the formation of space charge in the ferroelectric, and its redistribution at defects [35], and it can be a serious drawback for FE elements with pulse control. It has been shown [36,37] that slow relaxation phenomena (10–100 s) observed in ferroelectric elements in the paraelectric phase are caused by the presence of two types of charged defects in the film bulk: oxygen vacancies (~0.7 eV) and trap levels (~0.4 eV). It should be noted that oxygen deficiency in the film is accompanied by a distortion of the unit cell dimensions. Shallow trap levels, in turn, are determined by the structural imperfections of the film, namely, the presence of phases with different orientations, grain sizes, and the length of intergrain boundaries. Figure 6 shows the dependences of the response time parameter ∆C/C0 of capacitors formed on the basis of STO films on the strength of the control field. An analysis of Figure 6 allows us to conclude that the STO capacitors exhibit a tunability of 1.65 when a control field strength of 60 V/μm is applied, which is accompanied by a response time parameter of less than 4%. This result looks promising in comparison with the data published today, also presented in Figure 6. In [38], planar BST capacitors on an Al_2_O_3_ substrate were obtained, demonstrating a response time parameter of 10%, and in [33], STO capacitors on a silicon carbide substrate revealed ∆C/C0 of 7.5% in the same control field. The result obtained in this work is explained by the high structural quality of STO films and is a significant improvement in the level of capacitance non-return for FE planar structures.

Electrophysical measurements also provide indirect confirmation of the influence of oxygen vacancies on the film properties. Firstly, according to previous studies, oxygen vacancies influence the dielectric loss of the oxide film [39]. Specifically, the formation of an oxygen vacancy leads to the conversion of Ti^+4^ to Ti^+3^ and the emergence of hopping conductivity in the capacitor [40,41]. The presence of a peak corresponding to Ti^+3^ in the XPS spectrum indicates the presence of oxygen deficiency in the sample. It has been shown that an increase in the Ti^+3^ content in ferroelectric films is associated with a decrease in the oxygen concentration in the gas medium during film growth or annealing [42,43,44]. Secondly, the presence of oxygen vacancies directly affects the permittivity of the material and, consequently, its tunability [45]. Thirdly, the relaxation rate of the permittivity also depends on the presence of V_O2_ in the sample [46,47].

Thus, the planar capacitors obtained in this work (a) are realized on an accessible polycrystalline substrate with a permittivity of 9.8, with high mechanical and dielectric characteristics, without any anisotropy of properties; (b) demonstrate microwave Q-factors above 100 in the entire range of control voltages without deterioration of losses; (c) to the best of our knowledge, exhibit a response parameter that is the best of those published today for planar FE capacitors.

## 3. Experiment

Thin films of strontium titanate were obtained on alumina substrates by radio-frequency magnetron sputtering. A ceramic target of stoichiometric composition SrTiO_3_ with a diameter of 3” was made from a mixture of pre-synthesized chemically pure powders of SrCO_3_ and TiO_2_ (“Ferrite-Domain”, St. Petersburg, Russia). The films were synthesized at a substrate temperature of Ts = 900 °C. The choice of the deposition temperature of strontium titanate on an alumina is justified in [18]. A mixture of Ar:O_2_ in different proportions was used as the working gas during deposition. The deposition time of 500 nm thick films ranged from 2 to 6.5 h, depending on the composition and pressure of the working gas, while the pressure in the working chamber during deposition ranged from 3 to 10 Pa. The range of working gas pressures during the deposition of multicomponent films by RF magnetron sputtering is determined by the conditions of stable discharge combustion and an acceptable growth rate of the studied layers.

The crystal structure and phase composition of the films were controlled by X-ray phase analysis (XRD) using a DRON-6 diffractometer (Burevestnik, St. Petersburg, Russia) (λ = 1.54 Å). The recording regimes were as follows: Bragg–Brentano geometry, CuKα radiation (λ = 1.54 Å), Kβ filter, U = 30 kV, I = 20 mA, 2.5° Soller collimators, scanning range 2θ = 20–60°, scanning step 0.01°, and step exposure time 0.2 s, continuous mode. The crystal lattice parameters of the STO films were calculated from the angular positions of X-ray reflections, using reflections from the substrate as a reference.

The elemental composition and distribution of elements across the sample surfaces were studied using scanning electron microscopy (SEM) in low vacuum mode on an SM-32 Melytec scanning electron microscope (Zhejiang Nade Scientific Instrument Co., Ltd, Hangzhou, China). equipped with a thermionic tungsten cathode, backscattered and secondary electron detectors, and an Oxford Instruments energy-dispersive X-ray spectrometer (EDS, Abingdon, UK). The images were collected in backscattered electron detection (BSED) and secondary electron detection (ETD) modes at an accelerating voltage of 20–30 kV.

The surface morphology and roughness of the films were examined by atomic force microscopy (AFM) using an NTEGRA atomic force microscope (NT-MDT BV, Apeldoorn, The Netherlands) operated in semi-contact mode. Scanning was performed with silicon cantilevers HQ:NSC35/Al BS (MikroMasch, Wetzlar, Germany) with a resonance frequency of approximately 300 kHz, a spring constant of 4.8–44 N·m^−1^, and a tip radius of curvature of about 10 nm. AFM data processing and quantitative morphology analysis were performed using Gwyddion 2.69 software. For each sample, images were acquired at multiple random locations over scan areas of 3 × 3 μm and 10 × 10 μm. Prior to roughness parameter calculation, a first-order polynomial plane fit was applied to remove overall tilt from the data. Roughness values were calculated based on five measurement points per sample. Grain size analysis was performed using the ImageJ 1.54 software on 30 randomly selected locations for each sample.

The electrical properties of STO films were studied using planar capacitors with a gap width of 5 µm. Copper electrodes were deposited by thermal deposition using an adhesive chromium sublayer, followed by lithography and chemical etching. Capacitance C and quality factor Q = 1/tan δ measurements of the capacitors were performed at a frequency of f = 2 GHz using a half-wave stripline resonator and an HP 8719C vector analyzer (HP inc. Palo Alto, CA, USA). The measurement error of the transmission coefficient module did not exceed ±0.05 dB in the used range of signal levels. The resonator design provides an unloaded quality factor of 1000, which ensures a capacitance and quality factor measurement accuracy of 1 and 5%, respectively. A control voltage of up to 300 V was applied to the capacitor plates, providing a field strength in the capacitor gap of up to 60 V/μm. The tunability of the capacitors was calculated as the ratio of capacitances at zero and maximum applied control voltage *n* = C(0 V)/C(U_max_) in arb. units, and also as *n* = (C_max_ − C_min_)/C_max_, %. The response time parameter ∆C/C0 was estimated as the ratio of the capacitor capacitance 100 ms after removing the control voltage to the initial capacitance at zero bias voltage with measurement accuracy of 2%. For each sample under study, 5 capacitors were measured, after which the values of capacitance, quality factor, and response time were averaged. The optimal combination of interrelated nonlinearities and capacitor losses was determined using the commutation quality factor CQF.

## 4. Conclusions

Predominantly oriented strontium titanate films of high structural quality were successfully grown on alumina substrates using magnetron sputtering. The oxygen content in the gas mixture and the working gas pressure significantly affect the crystalline structure of the films and, consequently, their electrical properties. Planar capacitors based on (h00)-oriented STO films demonstrated tunability of *n* = 1.65 (40%), microwave Q-factors above 100 in the entire range of control voltages without deterioration of losses, and slow capacitance relaxation parameter significantly lower than currently published data for planar BST elements, opening up new possibilities for the use of strontium titanate films at microwaves. The obtained result of 4% ∆C/C0 at a field strength of 60 V/µm represents a significant reduction in the capacitance slow relaxation rate of FE capacitive planar structures and looks to bea promising result for use in devices with fast switching. The CQF value of 3300 for planar STO capacitors at microwaves has been achieved for the first time, which allows us to expect prospective characteristics of tunable microwave elements based on STO/alumina films.

## Figures and Tables

**Table 1:** EDS analysis data.

Element	Weight %	Sigma Weight %	At. %	Sigma At %
O	28.79	0.04	59.65	
Al	8.10	0.03	9.95	0.05
Ca	0.06	0.01	0.05	0.02
Ti	20.70	0.04	14.33	0.07
Sr	42.35	0.06	16.02	0.07
Total	100.00		100.00	

**Table 2:** Comparative electrical characteristics of capacitors based on STO films.

Substrate	Material	Design	E (V/µm)	tan δ (U0)	tan δ (U_max_)	*n* (%)	CQF (f)	Ref.
SmScO_3_	BST	planar	5	0.02	0.2	92	2750 (2 GHz)	[4]
SmScO_3_/SrRuO_3_	BST	MDM	10	0.33	0.09	98	2800 (100 kHz)	[5]
Alumina	STO	planar	60	0.009	0.014	46	3100 (1 GHz)	[18]
Sapphire/Pt	STO	4 electrodes	100	0.015	0.01	41	1730 (1 GHz)	[20]
STO:Nb/SrRuO_3_	STO	MDM	100	0.01	0.019	49	3300 (1 MHz)	[22]
SiC	STO	planar	50	0.008	0.009	36	2550 (2 GHz)	[33]
Sapphire/Pt	STO	4 electrodes	100	0.015	0.011	45	1921 (1 GHz)	[34]
Alumina	STO	planar	60	0.009	0.009	40	3300 (2 GHz)	This work

## Data Availability

The original contributions presented in this study are included in the article. Further inquiries can be directed to the corresponding author.
